# Altered expression of SIRPγ on the T-cells of relapsing remitting multiple sclerosis and type 1 diabetes patients could potentiate effector responses from T-cells

**DOI:** 10.1371/journal.pone.0238070

**Published:** 2020-08-27

**Authors:** Sushmita Sinha, Pranav S. Renavikar, Michael P. Crawford, Scott M. Steward-Tharp, Ashley Brate, Eva Tsalikian, Michael Tansey, Ezzatollah T. Shivapour, Tracey Cho, John Kamholz, Nitin J. Karandikar

**Affiliations:** 1 Department of Pathology, University of Iowa Health Care, Iowa City, Iowa, United States of America; 2 Department of Oral Pathology, Radiology and Medicine, College of Dentistry, University of Iowa, Iowa City, Iowa, United States of America; 3 Department of Pediatrics, University of Iowa Health Care, Iowa City, Iowa, United States of America; 4 Department of Neurology, University of Iowa Health Care, Iowa City, Iowa, United States of America; Uniformed Services University of the Health Sciences F Edward Hebert School of Medicine, UNITED STATES

## Abstract

Factors regulating self-antigen directed immune-responses in autoimmunity are poorly understood. Signal regulatory protein gamma (SIRPγ) is a human T-cell specific protein with genetic variants associated with type 1 diabetes (T1D). SIRPγ’s function in the immune system remains unclear. We show that T1D and relapsing remitting multiple sclerosis (RRMS) subjects have significantly greater frequency of rs2281808 T genetic variant, that correlates with reduced SIRPγ-expression in T-cells. Importantly, reduced SIRPγ-expression in RRMS and T1D subjects was not restricted to T variant, suggesting SIRPγ-expression is also regulated by disease specific factors in autoimmunity. Interestingly, increased frequencies of SIRPγ^low^ T-cells in RRMS and T1D positively correlated with proinflammatory molecules from T-cells. Finally, we show that SIRPγ^low^ T-cells have enhanced pathogenecity *in vivo* in a GVHD model. These findings suggest that decreased-SIRPγ expression, either determined by genetic variants or through peripherally acquired processes, may have a mechanistic link to autoimmunity through induction of hyperactive T-cells.

## 1. Introduction

Signal regulatory protein gamma (SIRPγ) is a human T-cell specific immunomodulatory protein encoded by the *SIRPG* gene [1, 2] with variants associated with autoimmunity in individuals with type 1 diabetes (T1D) in multiple GWAS studies [3–5]. Dysregulated *SIRPG* expression has also been demonstrated in other autoimmune conditions such as systemic lupus erythematosus (SLE) [6], suggesting that SIRPγ may play a critical role in immune dysregulation in multiple different autoimmune diseases. However, SIRPγ’s potential mechanistic contribution to autoimmunity remains unclear due to a knowledge gap regarding its function in the immune system. There are only a handful of studies that have tried to address the biological function of SIRPγ. Binding of SIRPγ with its ligand CD47 has been shown to facilitate cell adhesion [2, 7]. Engagement of SIRPγ on T cells by CD47 on APCs has been shown to enhance antigen-specific T-cell proliferation [2].

SNP rs2281808 TT is an intronic SNP present between exons 5 and 6 of *SIRPG* and causes a C/T variant. Multiple GWAS studies have shown that SNP rs2281808 is associated with type 1 diabetes (T1D) [3–5]. We recently showed that the rs2281808-T allele is associated with a reduction in SIRPγ expression on human T-cells leading to a hyperactivated state with lower activation threshold in healthy donors (HD) [8], suggesting that perturbation in SIRPγ levels may lead to immune dysregulation in individuals with autoimmune diseases.

In light of these findings, we determined whether the T allele and/or reduced SIRPγ expression is an underlying feature in two T-cell mediated autoimmune diseases, including relapsing remitting multiple sclerosis (RRMS) and type 1 diabetes (T1D), and if this can have pathogenic consequences, presumably due to exaggerated effector responses from SIRPγ low T-cells. We also asked whether CD4 and CD8 T-cell SIRPγ expression levels in these autoimmune diseases was an exclusive function of the carrier associated intronic SNP or if there were other relevant disease-specific factors that contributed.

## 2. Material and methods

### 2.1. Patients and control subjects

After obtaining informed consent, 19 type 1 diabetes (T1D) and 33 treatment naïve relapsing remitting multiple sclerosis (RRMS) patients were recruited at the pediatric endocrinology and neurology clinics, University of Iowa, respectively. De-identified leukoreduction buffy coat samples from 145 healthy donors (HD) were obtained from the University of Iowa DeGowin Blood Center, Department of Pathology. All studies were approved by the University of Iowa IRB according to Declaration of Helsinki principles. Mean age of HD, RRMS and T1D subjects was 52±14, 47±10 and 20±3 respectively. Lower age range in T1D cohort was due to the fact that only newly diagnosed T1D subjects were enrolled for the study. The M:F sex distribution in HD, RRMS and T1D was 80:65, 12:21 and 10:9 respectively. In a large correlation study between age, sex and SIRPγ expression in HD, we did not find any correlation between age and sex with SIRPγ expression.

### 2.2. Cell preparation and genotyping for rs2281808 detection

PBMC were isolated from buffy coats using Ficoll Hypaque (GE Healthcare Biosciences, Pittsburg, PA) density gradient. PBMC samples and sorted cells were stored in freezing media in liquid nitrogen until further use in multiple assays. DNA was isolated from PBMC samples using Qiagen mini DNA prep kit. Allelic discrimination PCR was done using TaqMan assay and probe as described previously [8].

### 2.3. Flow cytometric antibody staining

Anti-human antibodies used for extracellular multi-color flow cytometric analysis included: CD4-APC, CD8-BV786, SIRPγ-PE, CD3-Alexa700. Anti-human antibodies used for intracellular multi-color flow cytometric analysis included CD4- APC, CD8-BV786, SIRPγ-PE, CD3-FITC, IFNγ—Alexa700, TNFα PE-Cy7. All antibodies were obtained from either BD Biosciences (San Jose, CA), or Biolegend (San Diego, CA). PBMC samples were washed with 0.1% (w/v) sodium azide/ phosphate-buffered saline (Mediatech Cellgro) and stained with fluorescently labeled anti-human antibodies, then resuspended in 1% paraformaldehyde (Electron Microscopy Sciences, Hatfield, PA). Flow cytometric data were acquired on a 4-Laser LSRII using FACSDiva software (Becton Dickinson). Data were analyzed using Flow Jo (TreeStar, Ashland, OR). gMFI was used to look at the MFI of *SIRPγ*.

### 2.4. PBMC stimulation and cytokine detection

As described previously [9], one million cells from HD were stimulated with PMA/Ionomycin/Brefeldin for 6 hours. Cells were washed with 0.1% (w/v) sodium azide/ phosphate-buffered saline and stained intracellularly for detecting IFN-γ or TNF-α.

### 2.5. Induction of xGVHD in NSG mice

We used a graft versus host disease (GVHD) model to test whether SIRPγ^low^ T-cells display enhanced pathogenicity *in vivo* as compared to SIRPγ^high^ T-cells. From our pilot experiments, we determined that a minimum of 10 × 10^6^ SIRPγ^high^ T-cells are required to induce GVHD in NOD-SCID-gamma (NSG) mice. To test whether sub-optimal numbers of SIRPγ^low^ T-cells will be pathogenic in vivo, we transferred 6 × 10^6^ sorted SIRPγ^low^ or SIRPγ^high^ T-cells into NSG mice. The sample size of n = 3 in each group was based on the following power calculation: expected incidence of GVHD as 0% in SIRPγ^high^ vs 95% in SIRPγ^low^ recipients, false positive rate of 0.05% and 95% confidence interval. SIRPγ^high^ and SIRPγ^low^ T-cells were sorted from 3 different CC and TT healthy donors respectively using Miltenyi Biotech beads (Germany). The cell purity was >99% for each sample. Six million sorted cells from each CC and TT carrier were adoptively transferred into three different NSG mice and weight loss was monitored. At the end of the experiment, liver tissue was harvested, fixed in buffered formalin, paraffin embeded and H&E stained for histologic examination. Inflammation was scored as described previously [10].

### 2.6. Statistical analysis

The Chi-Square test was used to compare the rs2281808 genotype incidence between HD vs. RRMS and T1D patients and p<0.05 was considered significant. Data between the groups was analyzed with unpaired two-tailed Students *t*-test and p<0.05 was considered significant. One-way ANOVA with Tukey’s post-hoc test was performed to compare SIRPγ expression between the groups and p<0.05 was considered significant. Two-way ANOVA with Tukey’s post-hoc test was performed to compare SIRPγ expression between the genotypes and weight-loss in GVHD model and p<0.05 was considered significant. Correlation between SIRPγ^low^ T-cells and proinflammatory molecules was done using Pearson’s test and p<0.05 was considered significant.

## 3. Results

### 3.1. Significantly greater preponderance of T allele in multiple sclerosis and type 1 diabetes (T1D) patients

**Fig 1:**
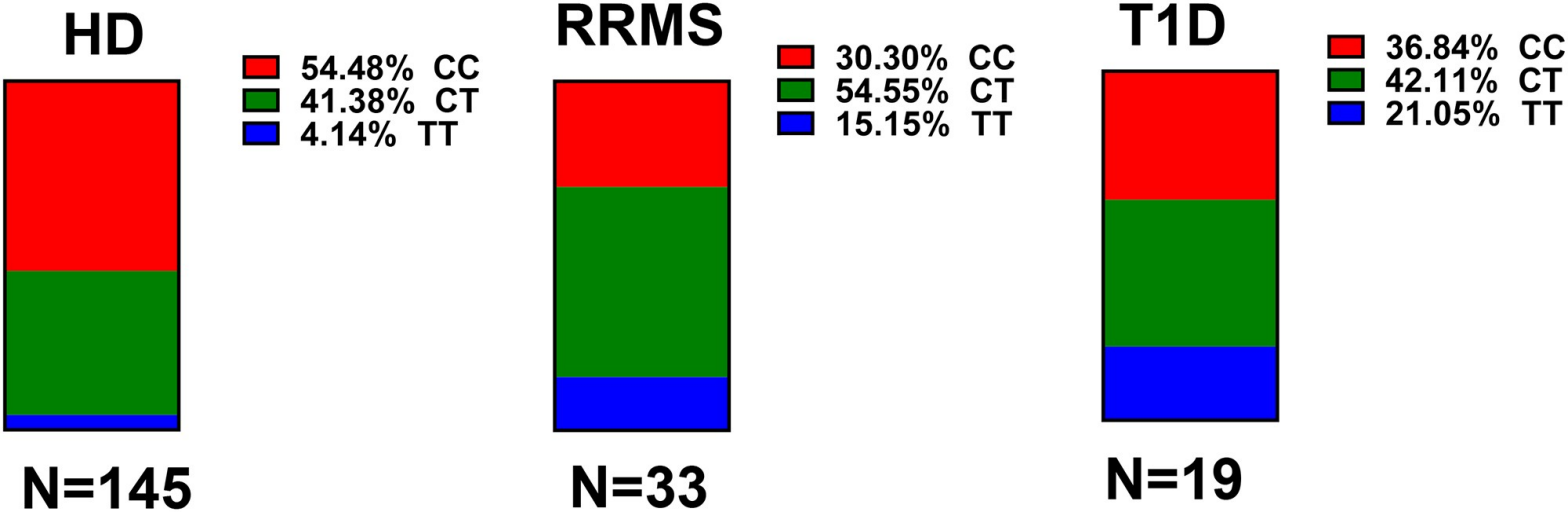
As compared to HD, RRMS and T1D patients show significantly greater preponderance of rs2281808 CT/TT carriers. DNA samples from healthy donors (HD), relapsing remitting multiple sclerosis (RRMS) and type 1 diabetes (T1D) were genotyped for SNP rs2281808 by TaqMan assay. Distribution of CC, CT and TT genotypes in study populations is shown in A. Comparison of CC, CT and TT genotype within the three study populations is shown in B. Both RRMS and T1D patients showed a significant preponderance of CT/TT carriers as compared to HD. Chi-Square test was used to compare the genotyping data and p<0.05 was considered significant.

Rs2281808 TT is an intronic SNP present between exons 5 and 6 of the *SIRPG* gene and causes a C/T variant. We have recently shown that the T allele is associated with hyperactivated T-cells with lower activation threshold in healthy donors (HD). Therefore, we asked whether the T allele may be overrepresented in two T-cell mediated autoimmune diseases, RRMS and T1D. Genotyping of 33 RRMS patients revealed that 10 (30%) and 18 (55%) patients showed CC and CT genotypes, respectively, whereas the TT variant was present in 5 (15%) patients (Fig 1). In T1D patients, 7 (37%) and 8 (42%) patients showed CC and CT genotype respectively, and the TT genotype was present in 4 (21%) patients (Fig 1). The rs22811808 genotype distribution in RRMS and T1D patients was different than HD where CC genotype was present in 55%, CT in 42% and TT was present in only 4% HD (Fig 1). While comparing the three genotypes in HD and RRMS patients, CC genotype was predominant in HD vs. RRMS (HD vs. RRMS; 55% vs. 30%, p<0.05). Interestingly, in RRMS subjects, CT and TT genotypes were significantly predominant vs. HD (HD vs. RRMS; CT: 41% vs. 55%, p<0.05; TT: 4% vs. 15%, p<0.05). Similarly in T1D subjects, CC genotype was predominant in HD vs. T1D (HD vs. T1D; 55% vs. 37%, p<0.05). While the CT genotype was not different between HD and T1D subjects, the TT genotype was significantly over-represented in T1D subjects (HD vs. T1D; TT: 4% vs. 21%, p<0.05). Collectively, the T allele (CT and TT) showed a significantly greater preponderance in both RRMS and T1D patients (T allele in HD vs. RRMS & T1D; 45% vs. 70% & 63%, p<0.05). Overall, our results show that the T allele of SNP rs2281808 in *SIRPG* is associated with two T-cell mediated autoimmune diseases, RRMS and T1D.

### 3.2. SIRPγ expression in autoimmunity is regulated outside of the rs2281808 genotype

**Fig 2:**
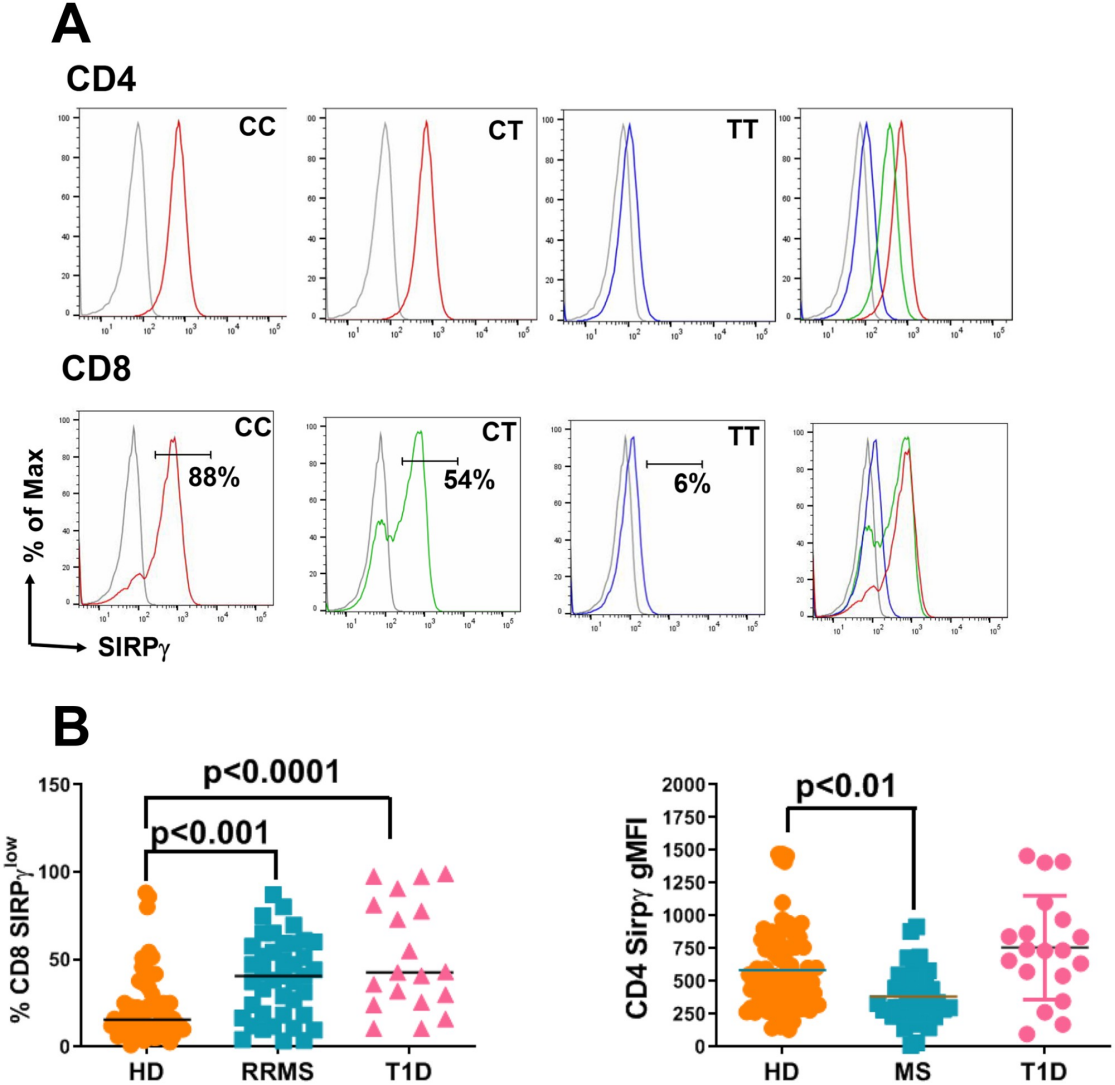
T-cells from RRMS and T1D patients have significantly reduced SIRPγ expression as compared to HD. Representative histograms for SIRPγ staining in RRMS patients is shown in A. Since SIRPγ shows a bimodal distribution on CD8 T-cells, SIRPγ^low^ vs. SIRPγ^high^ gates are shown for CD8 T-cells. Isotype control is shown in grey. PBMC samples from HD, RRMS and T1D patients were subjected to flow cytometry staining to detect SIRPγ on gated CD3, CD4 and CD8 T-cells as described previously [8]. SIRPγ gMFI data for CD4 T-cells and frequency of SIRPγ^low^ CD8 T-cells are shown in B. One-way ANOVA with Tukey’s multiple comparison was performed and p<0.05 was considered significant.

**Fig 3:**
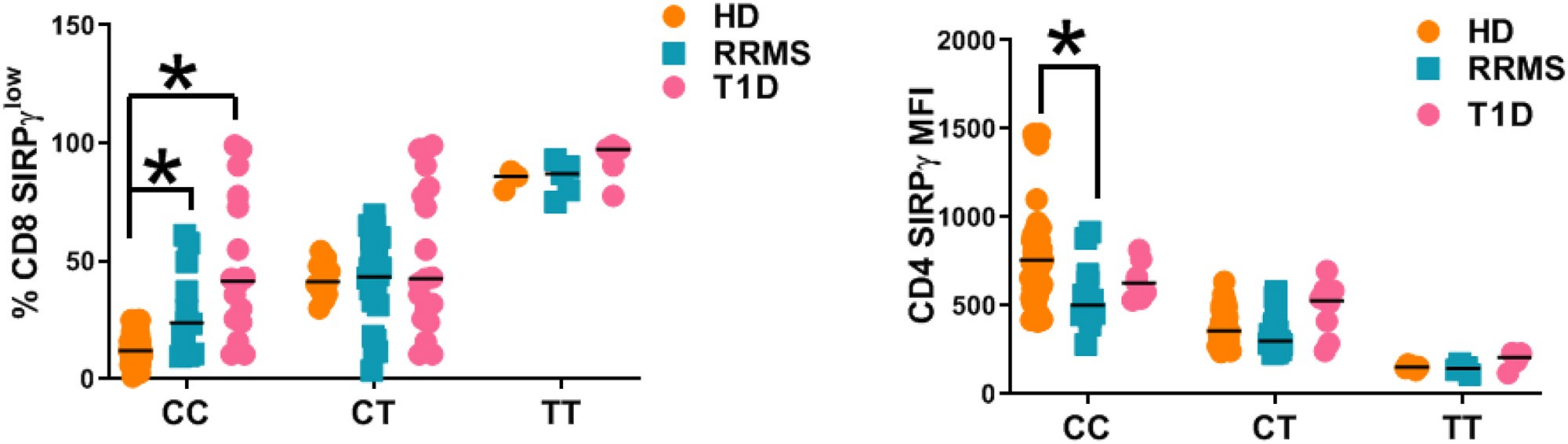
SIRPγ expression is regulated outside of rs2281808 genotype in autoimmunity. PBMC samples from HD, RRMS and T1D patients were subjected to flow cytometry staining to detect SIRPγ on gated CD3, CD4 and CD8 T-cells. SIRPγ expression on T-cells was compared between the rs2281808 genotypes in the study populations. SIRPγ gMFI data for CD4 T-cells and frequency of SIRPγ^low^ CD8 T-cells are shown. Two-way ANOVA with Tukey’s multiple comparison was performed and p<0.05 was considered significant.

SIRPγ staining on the T-cells of HD and T1D subjects has already been published before [8, 11]. Representative staining of SIRPγ on T-cells of RRMS subjects including low vs. high SIRPγ gates is shown in Fig 2A. We found that overall SIRPγ expression on T-cells of RRMS and T1D patients was significantly lower than T-cells from HD irrespective of the rs2281808 genotype. Both, RRMS and T1D patients had significantly greater percentages of CD8- SIRPγ^low^ T-cells as compared to HD (Fig 2B). Likewise, RRMS and T1D patients had significantly lower gMFI of SIRPγ on their CD4 T-cells as compared to HD (Fig 2B). Further analysis showed that the collective difference in SIRPγ expression between HD and autoimmune patients was driven by differences in SIRPγ expression on T-cells in CC carriers within the three groups. CC carriers from RRMS and T1D patients had significantly higher percentages of CD8- SIRPγ^low^ T-cells as compared to HD (Fig 3). Similarly, CC carriers from RRMS and T1D subjects had significantly lower SIRPγ -gMFI on CD4-T-cells as compared to HD (Fig 3). Therefore, reduced SIRPγ expression on T-cells of RRMS and T1D subjects as compared to HD was not solely attributable to an increased frequency of the rs2281808 TT genotype, suggesting regulation of SIRPγ by certain unknown disease-specific factors.

### 3.3. Increase in SIRPγ^low^ T-cells positively correlates with proinflammatory factors in RRMS and T1D patients

**Fig 4:**
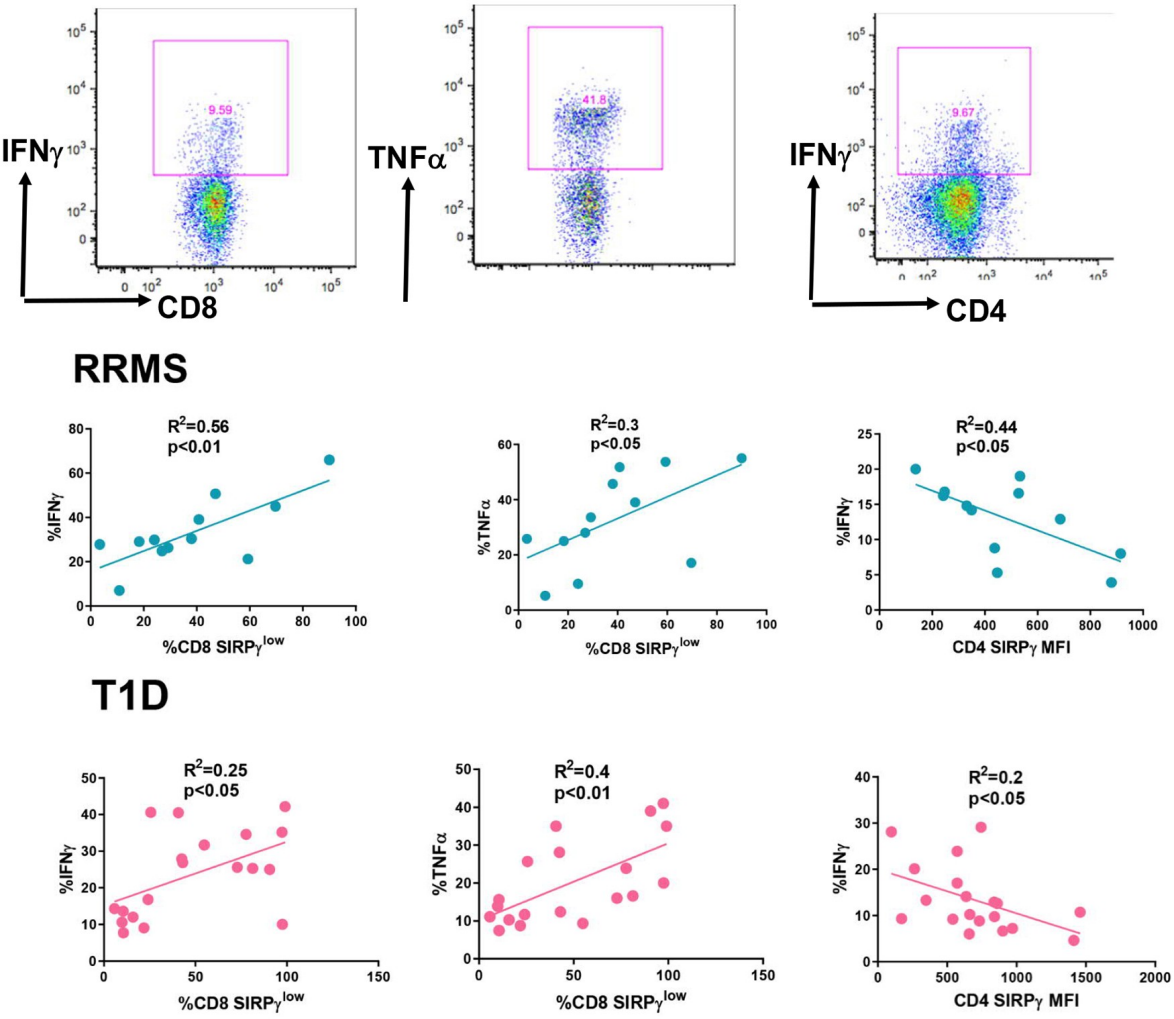
SIRPγ^low^ T-cells positively correlate with proinflammatory molecules in subjects with autoimmunity. PBMCs from RRMS and T1D patients were activated with PMA/INO in the presence of golgi plug for 6 hrs. Representative flow plots for IFNγ and TNFα staining on T-cells are shown. Following stimulation, cells were stained extracellularly with fluorescently tagged anti-CD3, CD4, CD8, and SIRPγ, followed by intracellular staining with anti-IFNγ, and anti-TNFα. Data was correlated with Pearson’s test and p<0.05 was considered significant.

We have previously shown that SIRPγ^low^ CD8 T-cells secrete greater amounts of effector molecules as compared to their SIRPγ^high^ counterparts [8]. Since we found that RRMS and T1D patients have significantly greater SIRPγ^low^ CD8 T-cells, we asked whether this increased frequency of SIRPγ^low^ T-cells might contribute to increased proinflammatory factors in an autoimmune setting. Indeed the percent of SIRPγ^low^ CD8 T-cells positively correlated with the percent of IFNγ and TNFα producing CD8 T-cells, both in RRMS and T1D patients (Fig 4). Likewise, IFNγ producing CD4 T-cells positively correlated with lower SIRPγ gMFI on CD4 T-cells in RRMS and T1D patients (Fig 4).

### 3.4. Sub-optimal numbers of SIRPγ^low^ T-cells are pathogenic in vivo

**Fig 5:**
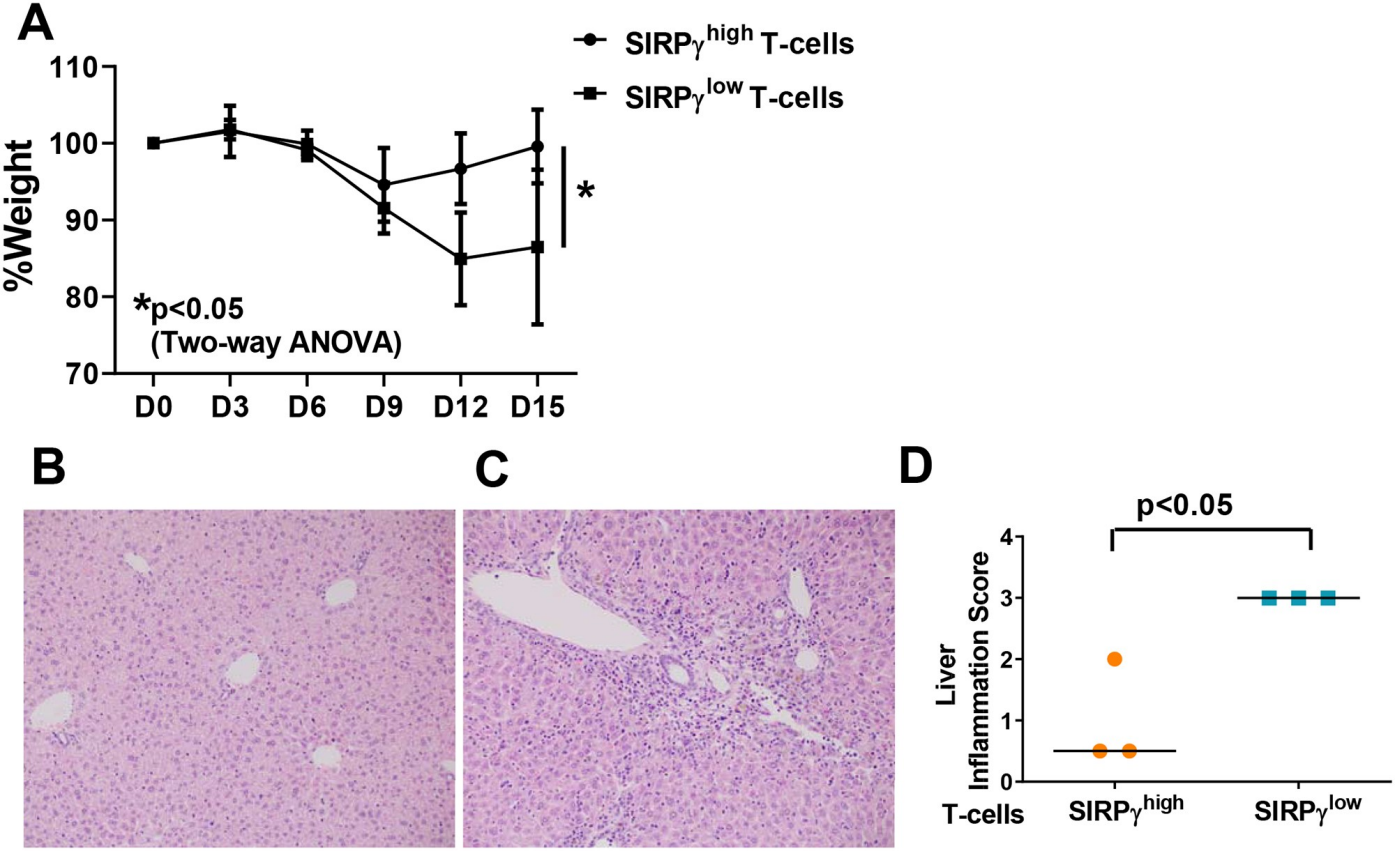
Unlike SIRPγ^high^ T-cells, sub-optimal numbers of SIRPγ^low^ T-cells are sufficient to cause xenoGVHD in NSG mice. Three million SIRPγ^high^ T-cells or SIRPγ^low^ T-cells, from 3 separate CC or TT carriers, were transferred into NSG mice. Weight was monitored as shown in A. Representative H&E staining on liver tissues is shown in B. Liver inflammation was scored by a pathologist blinded to the experimental grouping, using a previously published, established scale. Two-way ANOVA with Bonferroni’s multiple comparison test was performed to compare weight loss and p<0.05 was considered significant. An unpaired t-test was performed to compare inflammation scores and p<0.05 was considered significant.

We tested activation of SIRPγ^low^ vs. SIRPγ^high^ T-cells *in vivo* in the development of xenoGVHD in NSG (NOD-SCID-gamma) mice. This model system has been used to induce disease mediated by human CD4 T-cells [12–15]. Suboptimal numbers of SIRPγ high or low T-cells, from three different CC and TT HD, were transferred individually into three different NSG mice and weight-loss was monitored. Interestingly, all the three mice that received SIRPγ^low^ T-cells showed weight loss while all the SIRPγ^high^ T-cell- recipients remained healthy and exhibited no signs of GVHD (Fig 5A). Concomitantly, severe liver inflammation was detected only in the mice that received SIRPγ^low^ T-cells from TT carriers (Fig 5B & 5C). Liver sections from the mice that received SIRPγ^high^ T-cells from CC carriers were minimally involved with the exception of one mouse showing moderate inflammation. Importantly, no weight loss was detected in this mouse.

## 4. Discussion

The factors that exacerbate proinflammtory T-cell responses in autoimmunity are poorly understood. We have previously shown that reduced *SIRPγ* expression potentiates effector responses from human T-cells [8], suggesting that perturbed *SIRPγ* expression on T-cells may play a critical role in immune dysregulation of autoimmune diseases. The pathogenic effector role of T-cells, both in T1D and MS, is well established [16–32]. In both the diseases, autoreactive T-cells are thought to infiltrate the target organ and cause inflammation, leading to loss of insulin production (in T1D) or loss of nerve conduction (in MS). Here we report a novel association of reduced SIRPγ expression with two T-cell mediated autoimmune diseases including relapsing remitting multiple sclerosis (RRMS) and Type 1 diabetes (T1D). There is accumulating evidence in the literature to suggest that genetic variants in *SIRPγ* can lead to modulation of immune responses in humans. A recent study predicted that polymorphisms in *SIRPG* can interfere with transcription factors important in T-cell development [33]. A SNP in *SIRPG* was recently shown to be associated with the persistence of MenC-specific immunity following childhood immunization [34]. SNP rs2281808 in *SIRPG* has already been shown to be a risk factor for T1D by multiple GWAS studies [3–5]. Interestingly, early onset T1D patients provided more association evidence for rs2281808 [5]. We confirm the association of the rs2281808 TT genotype with T1D patients in our study population. Further, we found that RRMS patients also have a significantly greater preponderance of rs2281808 CT and TT allele as compared to healthy donors. We note that the difference in the prevalence of TT between RRMS vs. HD is smaller than that of T1D vs. HD. Therefore, this finding will benefit from corroboration in a larger sample size and we hope that this study will prompt investigators to study the prevalence of rs2281808 in other autoimmune diseases including RRMS.

We have recently shown that SIRPγ expression levels on CD4 and CD8 T-cells correlated with the genotype of the C/T polymorphism with expression being high in CC, intermediate in CT and low in TT subjects [8]. We found that this is also true for patients with RRMS and T1D. Additionally, we found that overall, SIRPγ expression on T-cells in patients with autoimmunity was significantly lower than healthy donors (HD), suggesting that SIRPγ expression may also be regulated by some as yet unknown disease-specific factors. Perturbed homeostasis of T-cells and constant exposure to pro-inflammatory cytokines in autoimmunity are plausible theories to be tested in future studies.

Positive correlation between SIRPγ^low^ T-cells and proinflammatory effector molecules from T-cells of RRMS and T1D patients suggest that reduced SIRPγ expression on T-cells could potentiate target organ-specific inflammation in autoimmunity. Indeed, we found that, unlike SIRPγ^high^ T-cells, suboptimal numbers of SIRPγ^low^ T-cells were enough to cause xGVHD in NSG mice. While the xGVHD experiment does not reflect the ability of T-cells to cause autoimmunity, it demonstrates that SIRPγ^low^ T-cells are hyperactive *in vivo* and can infiltrate target organs and cause inflammation. Since SIRPγ low vs. high T-cells even in the same individual produce significantly greater amounts of effector cytokines [8], we conclude that reduced SIRPγ expression functionally skews the T-cells toward potentiated effector responses. While CT and TT genotypes are associated with reduced SIRPγ expression on T-cells irrespective of the disease status, significantly reduced SIRPγ expression was seen selectively on the T cells of CC carriers in RRMS and T1D cohort. Our findings suggest that both rs2281808 genotype and as yet unknown disease specific factors are associated with significantly reduced SIRPγ expression on the T-cells of autoimmune subjects. Therefore, in future studies it will be more informative to look at SIRPγ expression on T-cells particularly in the disease cohorts. Future studies on the role of SIRPγ in immune regulation and dysregulation may enlighten our understanding of the targetable pathways involved in autoimmunity.
